# Reduced Circulating Endothelial Progenitor Cells and Downregulated GTCPH I Pathway Related to Endothelial Dysfunction in Premenopausal Women with Isolated Impaired Glucose Tolerance

**DOI:** 10.1155/2020/1278465

**Published:** 2020-01-25

**Authors:** Juan Liu, Xiangbin Xing, Xinlin Wu, Xiang Li, Shun Yao, Zi Ren, Haitao Zeng, Shaohong Wu

**Affiliations:** ^1^Department of Endocrinology, The First Affiliated Hospital, Sun Yat-sen University, Guangzhou 510080, China; ^2^Department of Gastroenterology, The First Affiliated Hospital, Sun Yat-sen University, Guangzhou 510080, China; ^3^Department of Traditional Chinese Medicine, The First Affiliated Hospital, Sun Yat-sen University, Guangzhou 510080, China; ^4^Department of Ultrasound, Guangzhou Development District Hospital, Guangzhou 510080, China; ^5^Department of Cardiology, The First Affiliated Hospital, Sun Yat-sen University, Guangzhou 510080, China; ^6^Center for Reproductive Medicine, The Sixth Affiliated Hospital, Sun Yat-sen University, Guangzhou 510080, China; ^7^Department of Ultrasound, The First Affiliated Hospital of Sun Yat-sen University, Guangzhou 510080, China

## Abstract

**Background:**

Individuals at a prediabetic stage have had an augmented cardiovascular disease (CVD) risk and CVD-related mortality compared to normal glucose tolerance (NGT) individuals, which may be attributed to the impaired vascular endothelial repair capacity. In this study, circulating endothelial progenitor cells' (EPCs) number and activity were evaluated, and the underlying mechanisms in premenopausal women with impaired glucose regulation were explored.

**Methods:**

Circulating EPCs' number and activity and flow-mediated dilation (FMD) were compared in premenopausal women with NGT, isolated impaired fasting glucose (i-IFG), or isolated impaired glucose tolerance (i-IGT). Plasma nitric oxide (NO), EPCs-secreted NO, and intracellular BH4 levels were also measured. The key proteins (Tie2, Akt, eNOS, and GTPCH I) in the guanosine triphosphate cyclohydrolase/tetrahydrobiopterin (GTPCH/BH4) pathway and Tie2/Akt/eNOS signaling pathway were evaluated in these women.

**Results:**

It was observed that the i-IGT premenopausal women not i-IFG premenopausal women had a significant reduction in circulating EPCs' number and activity as well as reduced FMD when compared to NGT subjects. Plasma NO levels or EPCs-secreted NO also decreased only in i-IGT women. The expression of GTCPH I as well as intracellular BH4 levels declined in i-IGT women; however, the alternations of key proteins' expression in the Tie2/Akt/eNOS signaling pathway were not observed in either i-IGT or i-IFG women.

**Conclusions:**

The endothelial repair capacity was impaired in i-IGT premenopausal women but was preserved in i-IFG counterparts. The underlying mechanism may be associated with the downregulated GTCPH I pathway and reduced NO productions.

## 1. Introduction

In the last decade, type 2 diabetes mellitus (T2DM) is regarded as one of the most popular metabolic diseases in China, which leads to an increased risk of diabetes-related morbidity or mortality due to its concomitant macrovascular and microvascular complications [1]. As a prelude to the development of T2DM, prediabetes is an intermediate state, characterized by isolated impaired fasting glucose (i-IFG), isolated impaired glucose tolerance (i-IGT), and combined IGT/IFG [2]. Researchers have suggested that prediabetes itself could be of a harmful state in which an increased risk of macrovascular and microvascular complications related with T2DM is already present [3–5].

The underlying mechanism of why some individuals with prediabetes are susceptible to microvascular and macrovascular complications is unclear yet. Multiplier effects may involve in the pathophysiology, wherein genetic susceptibility, activated protein kinase C (PKC), alterations in nitric oxide (NO) synthase, vascular endothelial growth factor, as well as a group of inflammation regulators may together contribute to such consequences [6–9]. Of them, more evidences have indicated that endothelial progenitor cells (EPCs), deriving from the bone marrow and attending vascular endothelium restoration, could modulate the cardiovascular (CV) function and angiogenesis and maintain endothelial homeostasis, thus contributing to vascular endothelial repair after the successional or concurrent blows of CV risk factors, for instance, hypertension, dyslipidemia, smoke, and hyperglycemia [10–12].

Indeed, in previous studies, the recruitment of EPCs was suppressed in both prediabetic patients [13] and women in gestational alterations of glucose tolerance [14], EPCs' declining number correlated with diminished vascular repair capability in both prediabetic [15] and diabetic patients [16]. However, so far, no study has been reported relating to the underlying mechanism of why the inability of circulating EPCs is present in prediabetic individuals. The question of whether or not there is a difference in the inability of circulating EPCs between i-IGT and i-IFG subjects is still left behind since the two different prediabetic status are regarded as having different pathophysiological characteristics.

The nitric oxide (NO), vascular endothelial growth factors (VEGFs), and granulocyte macrophage colony stimulating factor (GM-CSF) are important regulatory factors of circulating EPCs [17–19]. However, of them, only plasma NO and EPCs-secreted NO were observed to be associated with the alternations of endothelial improve capability in prehypertensive premenopausal women in our previous studies [20, 21].

Tie2/Akt/eNOS signaling pathway is a major upstream regulatory pathway of endothelial nitric oxide synthase (eNOS) [21]. We wonder if this pathway could involve the process of circulating EPCs' number reduction and dysfunction in prediabetic individuals. Previous studies have demonstrated that guanosine triphosphate cyclohydrolase (GTPCH I) deactivation could lead to tetrahydrobiopterin (BH4) insufficiency following with subsequent eNOS uncoupling [22–24], which may be attributable to the reduced eNOS-mediated NO production [17]. Thus, investigating the key protein expressions in the GTPCH/BH4 signaling pathway was also covered in the present study.

As previously studied, sexual difference in circulating EPCs' number and function was usually present among the middle-aged individuals because of the estrogen-related protective effect [20]. To exclude gender difference, the present study was conducted only in premenopausal women.

In summary, the following issues still need further investigation: (1) if the inability of circulating EPCs is present in premenopausal i-IGT or i-IFG women? (2) If that is, what is the possible underlying mechanism? Whether or not NO production will also be responsible for the deleterious changes of circulating EPCs in the targeted population just as it was in diabetic and hypertensive patients? Attempting to elucidate the abovementioned questions, the present study evaluated circulating EPCs' number and function, endothelial function in premenopausal NGT, i-IFG, or i-IGT women. In addition, plasma NO and EPCs-secreted NO were evaluated, and the key proteins in the guanosine triphosphate cyclohydrolase/tetrahydrobiopterin (GTPCH/BH4) pathway and Tie2/Akt/eNOS signaling pathway were evaluated meanwhile.

## 2. Materials and Methods

### 2.1. Characteristics of Subjects

Sixty-one premenopausal women were recruited and divided into three groups including NGT (*n* = 20), i-IFG (*n* = 21), and i-IGT (*n* = 20) according to their oral glucose tolerance test (OGTT) results. On the basis of Expert Committee on the Diagnosis and Classification of Diabetes Mellitus [25], subjects with fasting plasma glucose (FPG) < 100 mg/dl and the 2 h plasma glucose (2-h PG) after 75 g OGTT < 140 mg/dl are diagnosed with NGT, with 100 mg/dl ≤ FPG < 125 mg/dl and 2 h PG < 140 mg/dl were diagnosed with i-IFG, and with FPG < 100 mg/dl and 140 mg/dl ≤ 2 h PG < 200 mg/dl were diagnosed with i-IGT. The participants were excluded if they had the following conditions: established CVD, malignant disease, infectious or inflammatory disorders, smoke, polycystic ovary syndrome, previous hysterectomy or irregular menstrual cycles, pregnant or breastfeeding, with hormone replacement therapy, or any other use of E2/progesterone administration. The experimental protocol was approved by the Ethical Committee of our hospitals, and all the participants had signed the written informed consent for participation as enrolling in the present study.

Seventy-five-gram glucose solution was taken orally within 5 minutes after at least 8 hours of overnight fasting. Blood samples for glucose were collected before and at 30 min and 120 min postchallenge.

At the menstrual periods of the participants' menstrual cycles (day 2 to day 5 after the first day of menstrual bleeding), peripheral venous blood samples were drawn after overnight fasting. Serum total cholesterol (TC), triglycerides (TG), low-density lipoprotein cholesterol (LDL), high-density lipoprotein cholesterol (HDL), hyper-sensitivity C-reactive protein (hsCRP), glycosylated hemoglobin (HbA1c), and estradiol were also measured.

### 2.2. Evaluation of Circulating EPCs' Number

Flow cytometry and cell culture assay were used to assess the number of circulating EPCs. The detailed protocol has been written in the previous studies in assessing circulating EPCs' number [18, 26]. In brief, after the participants' peripheral blood mononuclear cells were separated by Ficoll density gradient centrifugation, they were cultured in endothelial cell basal medium-2 (EBM-2, Lonza Group, Ltd., Basel, Switzerland).

Following 7 days of culture, endothelial marker proteins were examined by flow cytometry. Peripheral blood (100 *μ*l) was incubated for 40 min at 4°C with phycoerythrin- (PE-) labeled monoclonal mouse anti-human antibodies recognizing cluster of differentiation (CD) 31, von Willebrand factor, and kinase-insert domain receptor or corresponding immunoglobulin G isotype control. Following this, erythrocytes were lysed, and the remaining cells were washed with PBS and fixed in 2% paraformaldehyde at 37°C for 10 min prior to further analysis using an ACEA NovoCyte^TM^. Cells were then incubated with monocytic lineage marker CD14, fluorescein isothiocyanate (FITC) anti-human CD45, and PE-Cy7 anti-human CD34 antibodies for 40 min at 4°C. NovoExpress software^TM^ was used to analyze the results.

The EPCs' counts were assessed by the ratio of CD34+KDR+ cells per 100 peripheral blood mononuclear cells (PBMNCs). The circulating EPCs were cultured for one week after isolation and quantified using DiI-acLDL uptake and FITC-labeled Ulex europaeus agglutin (lectin) staining which was similar as previous studies reported [26]. Two independent staff were assigned to count the cultured EPC which were identified as differentiating EPCs with DiI-acLDL/lectin double positive cells.

### 2.3. Migration and Proliferation Assay of EPCs

Circulating EPCs' migration was evaluated by employing a modified Boyden chamber which had been written detailly in our other studies [18, 20, 27].

In short, after being cultured for one week, 2 × 10^4^ EPCs were laid in the upper chamber of a modified Boyden chamber. DAPI was used to stain cell nuclei for quantification in 24 h. Cells which migrated into the lower chamber were counted manually in 3 random microscopic fields. EPCs' proliferation was evaluated by 3-(4,5-dimethylthiazol-2-yl)-2,5-diphenyltetrazolium bromide (MTT) (5 g/l; Fluka; Honeywell International, Inc., Shanghai, China) assay after being nurtured for 7 days.

### 2.4. Measurement of Flow-Mediated Dilation

Brachial artery FMD was measured according to the previous studies [28]. In short, high-resolution ultrasonography was used with a 5–12 MHz linear transducer on an HDI 5000 system (Washington, USA). The participants were supine for at least 15 min, and then their brachial arteries were scanned longitudinally 2 to 10 cm proximal to the antecubital fossa. After increasing the pressure in an upper-forearm sphygmomanometer cuff to 250 mmHg for 5 min and monitoring electrocardiogram after cuff deflation for 90 s, FMD was taken as the percentage augment in mean diastolic diameter after reactive hyperemia 55 to 65 s following deflation to baseline.

### 2.5. Measurement of NO, VEGF, and GM-CSF Levels in Plasma and Secretion by EPCs

Levels of NO, VEGF, and GM-CSF in plasma and secretion by cultured EPCs were measured as the previous study reported [18, 20, 26].

The present study measured nitrite in plasma with the Greiss method and presented the results as *µ*mol NOx of NO_3_^−^/NO_2_^−^ per liter of medium. Plasma levels of VEGF and GM-CSF were determined by high-sensitive ELISA assays (R&D Systems, Wiesbaden, Germany).

The cultured EPCs were transferred to Dulbecco's Modification of Eagle's Medium/20%-fetal bovine serum (Sigma-Aldrich; Merck KGaA) for 48 h, and the NO, VEGF, and GM-CSF levels were measured in the condition media with the similar methods as in the plasma.

### 2.6. Measurement of Intracellular Tetrahydrobiopterin and Western Blot Analysis

High-performance liquid chromatography with florescence detection was used to assess intracellular BH4 concentrations, which were calculated by subtracting BH2 and oxidized biopterin from total biopterins and expressed as p mol/mg protein [24, 29].

Measurements of the expressions of Tie2, Akt, eNOS, and GTPCH I were previously described [26]. Total proteins of EPC were harvested by cell lysis buffer (Cell Signaling Technology Inc, Danvers, MA, USA). Protein extracts were subjected to SDS-PAGE and transferred to polyvinylidene fluoride membranes (Cell Signaling Technology Inc.). The antibodies including rabbit anti-phosphorylated Tie2, anti-Tie2, anti-phosphorylated Akt, anti-Akt, anti-phosphorylated eNOS, anti-eNOS (1 : 1000; Cell Signaling Technology Inc.), anti-GTPCH I (1 : 1000; Santa Cruz Technology Inc.), and *β*-actin (1 : 1000; Santa Cruz Technology Inc.) were used. Proteins were visualized with HRP-conjugated anti-rabbit IgG (1 : 2000; Cell Signaling Technology Inc.), followed by use of the ECL chemiluminescence system (Cell Signaling Technology Inc.). The results were presented by the ratio of specific phosphorylated proteins to total proteins or total GTPCH I, Tie2, Akt, and eNOS proteins to *β*-actin and were statistically compared relative to those of premenopausal women with NGT.

### 2.7. Statistical Analysis

SPSS V11.0 statistical software (SPSS Inc., Chicago, Illinois, USA) was applied in statistical analysis. Quantitative variables of normal distribution were expressed as mean ± standard deviation (SD). ANOVA was applied to evaluate statistical significance, and the least significant difference post hoc test was used to compare the difference between each two groups (NGT vs. i-IFG, NGT vs. i-IGT, and i-IFG vs. i-IGT). Univariate correlations were assessed with Pearson's coefficient (*r*). It was considered as statistical significance when *P* values < 0.05.

## 3. Results

### 3.1. Baseline Characteristics

The demographic characteristics of individuals in this research are shown in Table 1. Age, BMI, lipid profile, hsCRP, and estradiol were comparable in the 3 groups. Compared with the NGT group, i-IFG or i-IGT women had an obviously increased HbA1c (both *P* < 0.05), i-IFG women had an increased FPG (*P* < 0.05), and i-IGT women had an increased 2 h PG (*P* < 0.05). I-IGT women had a higher HbA1c than i-IFG women (*P* < 0.05). FMD in i-IGT women was evidently lower than that in NGT or i-IFG counterparts (both *P* < 0.05). The difference in FMD was comparable between women with NGT and i-IFG (*P* > 0.05).

### 3.2. Circulating EPCs' Number and Activity in Three Groups

**Figure 1:**
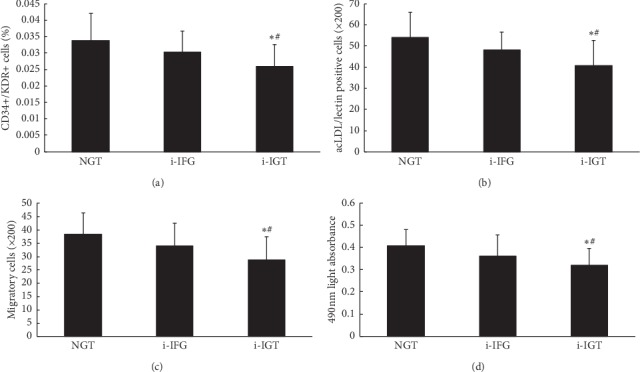
Number and activity of circulating EPCs in the three groups. Circulating EPCs were evaluated by (a) flow cytometric analysis and (b) cell culture assay. The number of circulating EPCs in premenopausal women with i-IGT was significantly lower than that in NGT and i-IFG women (both *P* < 0.05). No difference of the number of circulating EPCs was observed between NGT and i-IFG women. The migratory (c) and proliferative (d) activities of circulating EPCs in premenopausal women i-IGT were significantly decreased compared to NGT and i-IFG women (all *P* < 0.05). No difference of the activity of circulating EPCs was observed between NGT and IFG women. Data are given as mean ± SD (^*∗*^*P* < 0.05 vs. NGT; ^#^*P* < 0.05 vs. IFG, *n* = 20 for the NGT group and i-IGT group and *n* = 21 for the IFG group). NGT: normal glucose tolerance; i-IFG: isolated impaired fasting glucose; i-IGT: isolated impaired glucose tolerance; acLDL, acetylated low-density lipoprotein; CD, cluster of differentiation; KDR, kinase-insert domain receptor; EPCs, endothelial progenitor cells.

Premenopausal women with i-IGT had a significantly reduced number in circulating EPCs when compared to NGT or i-IFG women. The NGT group had a comparable EPCs' number with the i-IFG group (Figures 1(a) and 1(b)).

In a similar, EPCs' migratory and proliferative function were evidently reduced in the i-IGT group in comparison with the NGT or the i-IFG group (both *P* < 0.05). No difference in activity of circulating EPCs was noted between i-IFG and NGT women (Figures 1(c) and 1(d)).

### 3.3. FMD, NO, VEGF, and GM-CSF in the Plasma Levels and Secretion by EPCs in the Three Groups

**Figure 2:**
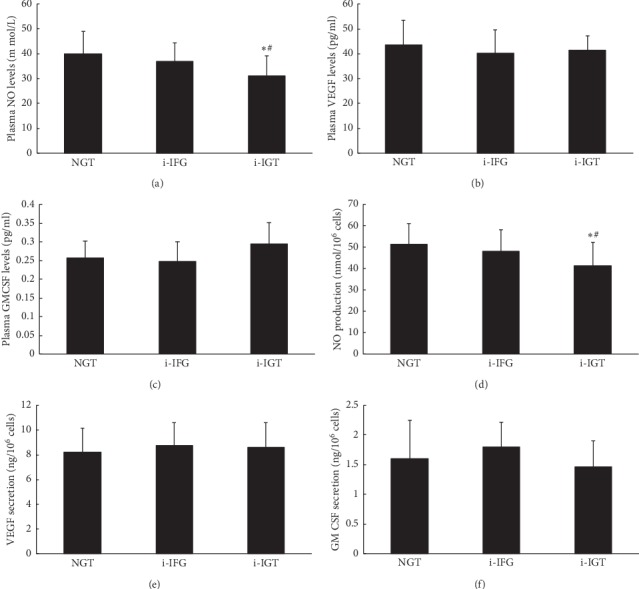
The NO, VEGF, and GM-CSF levels in plasma and secretion by EPCs in the three groups. Both the plasma NO level (a) and NO secretion by EPCs (d) in premenopausal women with i-IGT was significantly lower than NGT and i-IFG women (all *P* < 0.05). No difference of NO levels either in plasma or in culture media was observed between NGT and i-IFG women (a and b). No significant difference of VEGF or GM-CSF was found either in plasma level (b and c) or in secretion by EPCs (e and f) between the three groups. Data are given as mean ± SD (^*∗*^*P* < 0.05 vs. NGT; ^#^*P* < 0.05 vs. IFG, *n* = 20 for the NGT group and i-IGT group and *n* = 21 for IFG group). NGT: normal glucose tolerance; i-IFG: isolated impaired fasting glucose; i-IGT: isolated impaired glucose tolerance; GM-CSF, granulocyte macrophage-colony stimulating factor; NGT, normal glucose tolerance; NOx, nitric oxide; VEGF, vascular endothelial growth factor.

Difference between different glucose tolerant status concerning plasma NO levels was discovered, presenting that i-IGT women had a lower plasma NO levels compared to NGT women or i-IFG women (Figure 2(a)). No difference of the index was noted between i-IFG and NGT women. The differences in plasma VEGF and GM-CSF levels appeared to be insignificant between groups (Figures 2(b) and 2(c)).

EPCs-secreted NO had a similar trend as plasma NO levels had concerning the difference between the i-IGT and NGT or i-IFG group. NO secretion by EPCs only in i-IGT women was significantly lower than NGT women (Figure 2(d)). VEGF or GM-CSF secretion by cultured EPCs were comparable between these groups (Figures 2(e) and 2(f)).

### 3.4. Correlation between Circulating EPCs or NO Level and FMD

**Figure 3:**
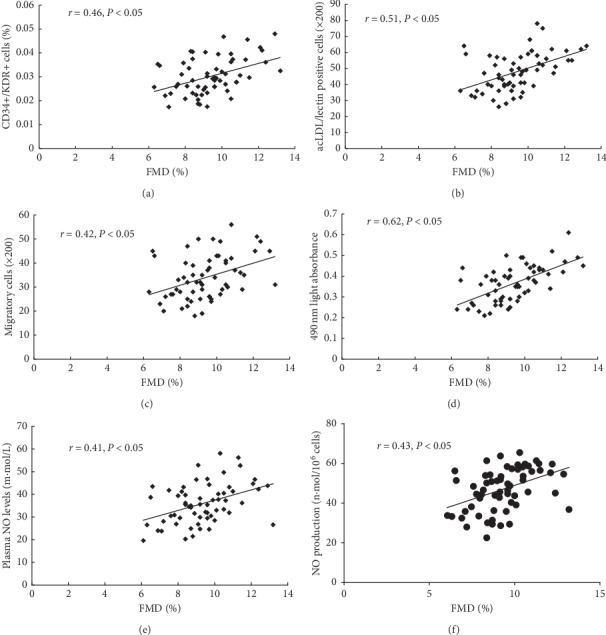
The correlations between circulating EPCs or NO production and FMD. The number of circulating EPCs evaluated by flow cytometric analysis (a) or by cell culture (b) strongly correlated with FMD. The migratory (c) or proliferate (d) activity of circulating EPCs also correlated with FMD. Both plasma NO level (e) and NO secretion by EPCs (f) correlated with FMD. LDL, low density lipoprotein; CD, cluster of differentiation; KDR, kinase-insert domain receptor; FMD, flow-mediated brachial artery dilatation; EPCs, endothelial progenitor cells; NO, nitric oxide.

Significantly positive correlations were observed between circulating EPCs' number and FMD (flow cytometry analysis, *r* = 0.46, *P* < 0.05, Figure 3(a)) (cell culture, *r* = 0.51, *P* < 0.05, Figure 3(b)), and FMD. The migration or proliferation of circulating EPCs also correlated with FMD (*r* = 0.42, *P* < 0.05, and *r* = 0.62, *P* < 0.05, respectively, Figures 3(c) and 3(d)). In addition, plasma NO levels (*r* = 0.41, *P* < 0.05, Figure 3(e)) as well as EPCs-secreted NO (*r* = 0.43, *P* < 0.05, Figure 3(f)) correlated with FMD.

### 3.5. Expression in GTCPH I Signaling Pathway and the Tie2/Akt/eNOS Signaling Pathway

**Figure 4:**
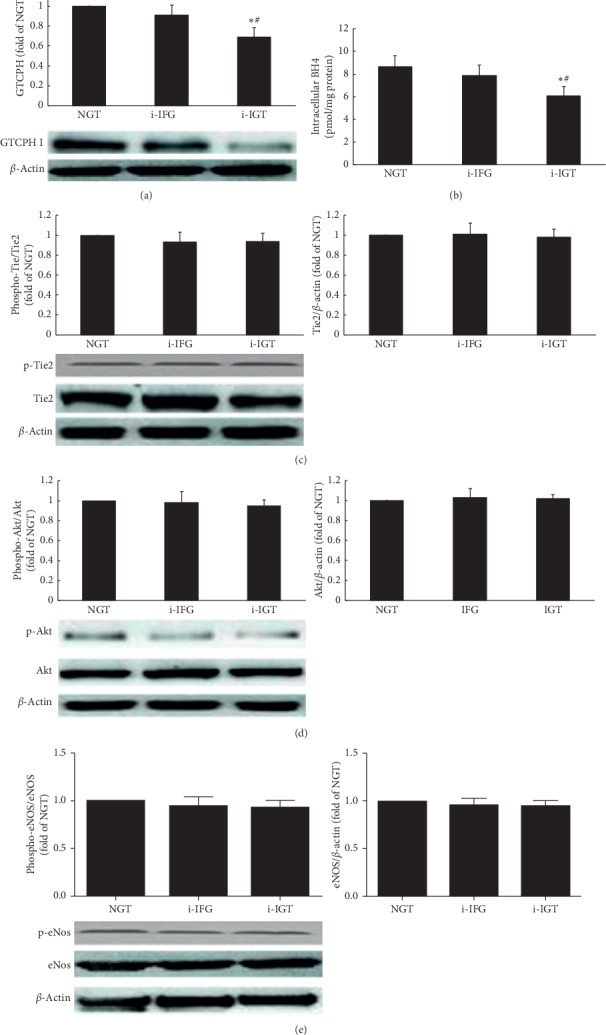
The CTCPH I/BH4 pathway and the Tie2/Akt/eNOS signaling pathway of circulating EPCs in the three groups. Both GTCPH I expression (a) and intracellular BH4 (b) levels were lower in premenopausal women with i-IGT than that in NGT and IFG women (all *P* < 0.05). No difference of the two indexes was observed between NGT and i-IFG women. Either the phosphorylation forms or the total protein of tie2 (c), Akt (d), and eNOS (e) of circulating EPCs had not exhibited significant difference between each two groups. Data are given as mean ± SD (^*∗*^*P* < 0.05 vs. NGT; ^#^*P* < 0.05 vs. IFG, *n* = 20 for the NGT group and i-IGT group and *n* = 21 for IFG group). NGT: normal glucose tolerance; i-IFG: isolated impaired fasting glucose; i-IGT: isolated impaired glucose tolerance; GTPCH: guanosine triphosphate cyclohydrolase; BH4: tetrahydrobiopterin; Tie2: tyrosine kinase with immunoglobulin and epidermal growth factor homology domain-2; Akt: protein kinase B; eNOS: endothelial nitric oxide synthase.

Since the malfunction of circulating EPCs of premenopausal women with i-IGT was presented, the key proteins' expressions both in GTCPH/BH4 signaling pathway or in Tie2/Akt/eNOS signaling pathway were assessed to explore its related mechanism. As shown in Figures 4(a) and 4(b), the expressions of GTCPH I and intracellular BH4 were lower in the i-IGT group than that in the NGT or i-IFG group but were at a similar level between the i-IFG group and NGT group.

No differences of the expressions of the phosphorylation or total tie2, Akt, and eNOS were observed between these 3 groups (Figures 4(c)–4(e)).

## 4. Discussion

The present results demonstrated that it was i-IGT premenopausal women that presented declined number and impaired function in circulating EPCs, which related with endothelial dysfunction. As another status of prediabetes, i-IFG premenopausal women had a well preservation of endothelial function and endothelial repair capacity relying on their comparable counts and function of circulating EPCs with NGT women.

Another interesting point in this study is the alterations in NO production that may at least partly account for the inability of circulating EPCs in i-IGT premenopausal women. Moreover, we further investigated the possible underlying mechanism for the reduced NO production, which was presented that downregulation of the GTCPH I/BH4 signaling pathway may contribute to the occurrence of this event. Therefore, we confirmed the previous results that the harmful effects of i-IGT on endothelial function as well as circulating EPCs in the certain population further explored its possibly underlying mechanism. The results could shed light on the cause of the increased risk of CVD in i-IGT population and highlight the need of taking effective actions to improve endothelial function in i-IGT premenopausal women.

Prediabetes is a particular metabolic state between the normal glucose metabolism and diabetic hyperglycemia. There is a high risk for the progression of prediabetes to diabetes with an estimated 10% of annual conversion rate [30]. In addition, patients in the prediabetic state, especially in the i-IGT state, have had already increased risk for a scope of microvascular and macrovascular complications [5, 6].

Many evidences have concentrated on the impairment in circulating EPCs of patients with diabetes [31]; however, a few of studies were conducted to explore the changes in circulating EPCs in prediabetes. Nathan et al. observed attenuated circulating EPCs in Asian Indian prediabetic men [32]. Fadini et al. observed an obvious reduction of circulating EPCs in IGT subjects and newly diagnosed T2DM patients [33]. Similar results were also observed in obese/overweight IGT subjects [34] and gestational women with IGT [14]. As was consistent with these previous studies, we also noted the reduction and impaired functional activity of circulating EPCs in i-IGT although in a different population that is relatively younger and premenopausal, which confirmed the inability of circulating EPCs preceded overt diabetes that cut across different populations and ages.

Endothelial dysfunction is one of the key indicators and contributors for CVD, also involving in the pathogeneses of macrovascular complications of T2DM [35]. FMD, which is usually used to assess vascular endothelial dilation function [28], was lower in i-IGT premenopausal women than age-matched NGT women and positively correlated with circulating EPCs' number as well as function. Thus, it could be hypothesized that the inability of circulating EPCs had a deleterious effect on endothelial function and may augment CVD-related risk in prediabetes.

Although i-IFG as well as i-IGT are classified into impaired glucose regulation preceding overt diabetes, there is a controversy over their risk of causing CVD [2, 36]. In fact, it seems to be that CVD may be more related with postchallenge glucose than fasting glucose, which may be explained by differences in metabolic traits, for example, insulin secretion and insulin sensitivity and other CVD risk factors between i-IGT and i-IFG subjects [37]. It is usually acknowledged that i-IGT, characterized by the elevation of postload glucose during OGTT and normal FPG, is largely related with defect of the first-phase insulin secretion and peripheral insulin resistance (IR), while i-IFG, characterized by moderately increased FPG and normal postload glucose, is mainly caused by increased hepatic glucose production resulting from hepatic IR [37].

In previous studies, there seemed to have a contradictory phenomenon about the alternations of endothelial function and circulating EPCs in i-IFG individuals. Subjects with i-IFG seemed to maintain a normal level of circulating EPCs in the study of Fadini et al. [33]; however, in other studies, endothelial dilated function was reported to be declined, which could be improved by regular aerobic exercise training [38, 39]. We did not observe either the inability of circulating EPCs or impairment of endothelial dilated function in i-IFG premenopausal women. The divergence may be able to be explained by the enrolled populations with an obvious age difference (with an average 65 years in previous studies vs. 42 years in the current study) and only in premenopausal state in the present study. So it could be speculated that relatively high estradiol levels in premenopause may exert a protective effect on endothelial function in women with IFG although the relationship of the estradiol level and FMD in these patients needed to be further elucidated.

The reason of why there is a difference in the alternations of circulating EPCs between i-IFG and i-IGT premenopausal women is unclear yet. We speculate that the relatively constant postload glucose in i-IFG subjects could be counted on for the difference since glucose excursion could lead to a greater degree of reduced EPCs compared to constantly high glucose concentrations in some vitro experiments [40]. We might hypothesize that the preservation of endothelial function could be partly attributable to the relatively low risk for CVD in i-IFG population.

Though many studies had observed the inability of circulating EPCs in i-IGT population, the exploration of mechanism about this phenomenon does not go far enough for now. Many experiments have suggested that hyperglycemia could lead to dysfunction of circulating EPCs by acting against their production and promoting their removal from the circulation [35, 41, 42]. Among these classical regulators of the insufficiency and/or dysfunction of circulating EPCs such as NO, VEGF, and GM-CSF [42, 43], and only NO production seems to be more likely to be responsible for the changes in circulating EPCs in many clinical or subclinical situations including obesity, smoke, prehypertension, and diabetes [11, 21, 24, 44]. As expected, NO production could also be attributed to the changes in circulating EPCs in i-IGT premenopausal women. Decreased NO levels both in plasma and secretion by EPCs in this certain population were observed, and positive correlation between NO production and the number or functional indexes of circulating EPCs in all populations was found in the present study.

Similarly, the underlying mechanism that hypeglycemia could contribute to the depletion or dysfunction of EPCs is closely related with NO production and NO bioavailability [41]. In in vitro experiments, high glucose concentrations could reduce intracellular BH4 cofactor (tetrahydrobiopterin), thus uncoupling endothelial NO synthesis (eNOS) and reducing NO bioavailability [42]. In the current study, we also observed the reduced expression of GTCPH I and decreased intracellular BH4 levels in i-IGT premenopausal women that was in parallel with the reduced NO production, indicating that the reduced expression of the two key proteins may contribute to the insufficiency of NO production either in the plasma or secretion by EPCs. Therefore, based on these results, we could speculate that the insufficiency and/or dysfunction of circulating EPCs in i-IGT premenopausal women may be associated with the decreased NO production through downregulating GTCPH I/BH4 signaling pathway.

Tie2/Akt/eNOS signaling pathway is also important in modulating circulating EPCs' number and function [45]. As previously demonstrated, the expressions of the phosphorylation of several key proteins in this signaling pathway including tie2, Akt, and eNOS, in circulating EPCs, were reduced in prehypertensive premenopausal women with DM [21]. However, in the present study, we did not observe any reduced expression of either the abovementioned proteins themselves or their phosphorylation forms in i-IGT premenopausal women, indicating that decreased NO secretion by EPCs could be independent of the Tie2/Akt/eNOS pathway in i-IGT premenopausal women.

Several implications can be taken from the current results. First, in premenopausal women with i-IGT but not with i-IFG, circulating EPCs' number and function declined, which was related to endothelial dysfunction. The results may partly explain the difference of the risk of CVD in the two prediabtic status. On the contrary, proper intervention to enhance vascular repair capacity should be considered in premenopausal women when they are at a prediabetic (especially IGT) stage. Second, we did not observe the difference in endothelial function and circulating EPCs between i-IFG and NGT premenopausal women, indicating that impaired fasting glucose metabolism alone may be not enough to impair endothelial function. Third, the reduced number or activity of circulating EPCs may be associated with the decreased NO production, which was at least partly mediated by the GTPCH I/BH4 signaling pathway. The clinical interventions aiming to increase the NO production such as regular exercise, quitting smoking, and statins usage may be more effective in improving endothelial dysfunction of i-IGT premenopausal women.

## 5. Conclusions

The present investigation demonstrated the unfavorable effects of i-IGT on circulating EPCs and endothelial function in premenopausal women, which could correlate with NO production reduction as well as downregulation in the GTPCH I/BH4 signaling pathway. Endothelial function and circulating EPCs' number or activity were preserved in i-IFG premenopausal women, indicating that moderately increased fasting glucose in premenopausal women may not exert much negative effect on endothelial function. Our results provided new vision to vascular preservation in i-IGT premenopausal women, pointing out that the GTPCH I/BH4 signaling pathway could be a latent point for improving endothelial improve capacity.

## Figures and Tables

**Table 1:** Clinical and biochemical characteristics.

Characteristics	NGT (*n* = 20)	i-IFG (*n* = 21)	i-IGT (*n* = 20)
Age (years)	40.1 ± 5.3	41.2 ± 4.8	43.9 ± 3.6
Height (cm)	162.0 ± 7.6	163.1 ± 6.5	161.2 ± 5.9
Weight (kg)	61.2 ± 5.0	60.6 ± 6.9	61.9 ± 4.9
Body mass index (kg/cm^2^)	23.4 ± 2.1	22.8 ± 2.5	23.9 ± 1.8
Systolic blood pressure (mmHg)	121.2 ± 11.4	123.4 ± 7.8	121.9 ± 10.0
Diastolic blood pressure (mmHg)	71.7 ± 6.5	73.2 ± 5.4	70.7 ± 7.2
Heart rate (beats/min)	71.0 ± 6.3	73.8 ± 8.1	75.7 ± 8.7
Aspartate transaminase (mmol/L)	27.2 ± 6.1	25.5 ± 4.7	23.2 ± 4.6
Alanine aminotransferase (mmol/L)	25.6 ± 8.0	23.7 ± 4.6	22.6 ± 4.8
Blood urea nitrogen (mmol/L)	5.2 ± 1.3	5.0 ± 1.0	5.5 ± 0.9
Creatinine (mmol/L)	62.2 ± 19.0	60.6 ± 14.5	65.2 ± 15.7
Low-density lipoprotein (mmol/L)	2.80 ± 0.46	2.87 ± 0.44	2.99 ± 0.45
Total cholesterol (mmol/L)	4.80 ± 0.54	4.90 ± 0.56	5.04 ± 0.51
High-density lipoprotein (mmol/L)	1.43 ± 0.25	1.39 ± 0.24	1.35 ± 0.24
Triglyeride (mmol/L)	1.37 ± 0.22	1.42 ± 0.21	1.45 ± 0.20
Fasting plasma glucose (mmol/L)	4.81 ± 0.45	6.18 ± 0.38^*∗*^	4.96 ± 0.35
2-hour plasma glucose (mmol/L)	6.51 ± 0.62	7.14 ± 0.45	9.53 ± 1.04^*∗*^^#^
Glycosylated hemoglobin A1c (%)	5.30 ± 0.60	5.76 ± 0.49^*∗*^	5.91 ± 0.42^*∗*^^#^
Hypersensitive C-reactive protein (mmol/L)	1.15 ± 0.66	1.32 ± 0.71	1.44 ± 0.86
Estradiol (pmol/L)	237.4 ± 33.5	215.9 ± 37.4	202.1 ± 23.6
Flow-mediated brachial artery dilatation (%)	11.0 ± 1.41	9.63 ± 1.33	8.09 ± 1.36^*∗*^^#^

*Abbreviations*. NGT, normal glucose tolerance; i-IFG, isolated impaired fasting glucose; i-IGT, isolated impaired glucose tolerance. *Notes.* Data are given as mean ± standard deviation. ^*∗*^*P* < 0.05 vs. NGT; ^#^*P* < 0.05 vs. i-IFG.

## Data Availability

The datasets during the current study are accessible from the corresponding author on reasonable request.
